# Consolidation of *Aedes albopictus* Surveillance Program in the Autonomous Community of the Region of Murcia, Spain

**DOI:** 10.3390/ijerph17114173

**Published:** 2020-06-11

**Authors:** Francisco Collantes, Manuel José Méndez, Caridad Soto-Castejón, Eva María Muelas

**Affiliations:** 1 Departamento de Zoología y Antropología Física, Facultad de Biología, Universidad de Murcia, 30100 Murcia, Spain; 2 Servicio de Sanidad Ambiental, Consejería de Salud de la Región de Murcia, 30008 Murcia, Spain; manuelj.mendez@carm.es (M.J.M.); caridad.soto@carm.es (C.S.-C.); evam.muelas@carm.es (E.M.M.)

**Keywords:** entomological surveillance, regional program, *Aedes albopictus*, Spain

## Abstract

Background: Due to the Spanish legal framework, the national program for vector-borne diseases results from the agreement between national and regional governments, and it is the basis for the development of the regional programs, which should include the regional entomological surveillance program. *Aedes albopictus* was recorded for the first time in the Region of Murcia, in 2011. It gave rise to a new epidemiological scenario due to the presence of a competent vector of several arboviruses, which resulted in autochthonous cases of dengue in 2018. Methods: 40 out of 45 municipalities participated in the regional entomological surveillance program, and 266 sampling points were established, with two ovitraps at each site as pseudo-replications. The study period was from April 16th to November 26th, with bi-weekly sample collections: 16 regional samplings were carried out. Results: Regional participation was high, and data loss was low (1.26%). *Ae. albopictus* was detected in 4.9% of samples and 89.4% of points, located in 39 of the 40 municipalities. The intensity of the presence of *Ae. albopictus* was estimated by a positivity index, that is, the percentage of positive samples over time. The vector phenology was obtained at a regional level, using the number of eggs as estimation of population density and the positivity values of points and municipalities. Every two weeks, real-time results were provided to the municipalities, which could use them as a vector management tool. Conclusion: The regional entomological surveillance program for *Ae. albopictus* in the Region of Murcia was consolidated in 2019, with standardized and comparable methods. Almost all the municipalities of the region have observed the presence of *Ae. albopictus*, although intensity and spatial and temporal cover vary among them.

## 1. Introduction

There are no effective prophylactic measures for several arboviral diseases transmitted by *Aedes* Meigen, 1818 mosquitoes, such as dengue, chikungunya or Zika fevers [1,2]. Thus, prevention strategies for these diseases are almost limited to vector control and the avoidance of bites.

In 2018, the first autochthonous cases of dengue fever were detected in Spain (five of them in the Region of Murcia, all belonging to the same viral strain, identified by PCR [3]). They could be considered as the last cases of dengue since the 20th century [4]. These recent cases are associated with *Aedes albopictus* (Skuse, 1895), while the former ones corresponded to summer arrivals of *Aedes aegypti* (L., 1762) [5]. This assertion is based on the fact that *Ae. aegypti* has not been detected in peninsular Spain since 1953 [6], whereas the broad range of distribution of *Ae. albopictus* in the areas involved [7,8] and dengue-infected specimens of *Ae. albopictus* by dengue virus had already been found in Spain in 2015 [9].

Spain is not a federation, but it has a devolved political system, and Spanish regions, known as autonomous communities, are responsible for health management as a shared devolved power. Therefore, pursuant to the Spanish legal framework, the Ministry of Health cannot develop a national plan for vector-borne diseases, but, in contrast, it is an agreement with and between the regional governments. National and regional representatives meet in the Inter-Territorial Council of the National Health System, to reach consensual agreements such as the *Spanish National Program for Vector-Borne Diseases, Part I: Dengue, Chikungunya and Zika* (which was promoted by the Spanish Ministry of Health) [10]. The national program document includes a fully comprehensive approach to these three diseases: etiology, epidemiology, epidemiological surveillance, competent vectors, risk evaluation, risk scenarios and prevention and control recommendations. Due to the diversity of the issue, many health-related professionals are involved: doctors, epidemiologists, environmental health professionals (in Spain, pharmacists or veterinaries), entomologists, pest control technicians, etc.

In this legal framework of devolved powers, the document resulting from the agreement represents the starting point for the regions to develop their own programs. The most recent compilation of the state of the issue is compiled in the entomological surveillance report of 2018 [11] of the Spanish Ministry of Health, which establishes the Spanish regions which officially have their own program: Basque Autonomous Community, Catalonia, Community of Madrid, Community of Navarre, Region of Murcia, Valencian Community and La Rioja. In some regions, plans were in development, while others had no program at all. In these regions, some municipalities were surveyed as municipal research initiatives. In 2019, some other regions, such as Aragón, Castilla-La Mancha and the Principality of Asturias, started their programs (S. Delacour, pers. comm.). In Andalusia, the sampling processes were carried out by the University of Murcia, until 2018, as an outsourced service for the University of Zaragoza, which was in charge of the Ministry of Health project “Entomological surveillance in airports and ports against imported vectors of exotic infectious diseases, and surveillance of potential native vectors of such diseases”. In 2019, the study was interrupted because this autonomous community was not interested in its continuation and rejected assuming all or part of the sampling cost.

*Ae. albopictus* was recorded for the first time in the Region of Murcia, in 2011 [12]. Subsequent detections were made in municipal or regional studies [7,8,13,14,15], and the first outdoor winter reproductive population in Europe was recorded [16] here. The regional entomological surveillance program in the Autonomous Community of the Region of Murcia began in 2018; however, several irregularities with regard to standardization were observed. The participation period was not the same in all municipalities, and sampling synchronization was deficient. In addition, other associated aspects were not developed, such as the protocols for the cases of arboviruses, with respect to site inspection and vector control thereof.

The objectives of the program are to estimate the health risk (both vector-borne diseases, as well as biting nuisance) and serve as a tool to facilitate municipal control of mosquitoes. Therefore, we tried to cover the max of urban zones and to keep an appropriate level of entomological surveillance, according to workforce availability.

The aim of this paper is to present the consolidated organization of the regional program for entomological surveillance of *Ae. albopictus* in the Region of Murcia. The results of 2019, besides their intrinsic value as data, also serve to illustrate the process of program development and implementation.

## 2. Materials and Methods

### 2.1. Study Area

The Region of Murcia is located in the southeast of Spain, on the Mediterranean Coast. It consists of a single province (area 11,313.9 km²) and 45 municipalities (fewer than in other parts of Spain, because the majority of them are bigger than usual). The population density is higher (132 pop./km²) (total population in 2019 was 1,493,898 people) than the Spanish average (92.11 pop./km²) [17]. Due to these characteristics, some municipalities usually contain several urban areas.

Based on previous sampling points, the coverage in the different urban areas of the 45 municipalities in the region was reviewed, since the placement of traps in these kinds of environments (urban/suburban) was the main criterion for eligibility. In total, 278 sampling points were proposed to the 45 municipalities, although proportion per municipality varied due to the differences in area size and urban geography. The municipality of Murcia made its participation conditional on the reduction of the number of points, from the 71 initially proposed to 58 points, which made up its own preexisting program. In the sampling 7th, one point more was placed in the municipality of Cieza, after numerous citizens’ complaints of mosquito bites. Thus, the program finally included 266 points (Table 1) which cover a high percentage of the regional urban areas.

Google Earth and Street View were used to pre-georeference all sampling points. Google Earth allows the estimation of the traps-covered area, and Street View provides very accurate and updated images of the Spanish territories, so the placement of gardens, big tub pots, plots with vegetation, bushes, hedges, etc., observed is very similar to the current state. This previous work saves fieldwork, and it is essential when the personnel are not expert. The points were codified by using the three letters from the municipality’s name and a two-digit number. Proposed coordinates for sampling points were supplied to municipal representatives. The technicians rectified them on the ground, if necessary, when ovitraps were placed, and definitive coordinates were given back.

Sampling points were not moved in the study period, with the exception of two points in the municipality of Los Alcázares.

### 2.2. Sampling Method, Samples Study and Taxonomic Identification

Surveillance implies covering wide areas and repetitive sampling (every one or two weeks) for an extended period (a year or, at least, a biological season). Ovitraps are frequently used for the surveillance of container mosquitoes because of their low cost and their potential for large-range coverage, and also because they are a better option than larval sampling to detect positive areas of *Ae. albopictus*/*Ae. aegypti* [18,19,20,21]. Therefore, we concluded that this method was the best way to standardize sampling in the regional study, based on its recognized efficiency, efficacy and cost-effectiveness.

Thus, ovitraps with hardboard paddles (HDF) were the sampling method used. Traps were baited, as an attractant, adding dry granulated rabbit food based on alfalfa [22]. Two traps were placed at each point as pseudo-replication, separated by a distance of 2 to 30 m (at greater distances, they could act as different points). As females could spread eggs between them due to their proximity [23], these two samples were not independent but paired samples and were considered as a single one. Based on our previous experience, the use of pseudo-replication reduces information loss in every point (due to the disappearance of paddles) and decreases false negatives (derived from non-perceptible wrong emplacement of the trap, e.g., under a plant which has not been identified as a repellent). Each paired sample was identified differently (trap A or B), in order to detect possible errors, as explained in the statistical methodology. According to its own directive, the municipality of Murcia only established one ovitrap per point.

The municipalities were responsible for collecting the samples and replacing the paddles, so personnel diversity was significant: municipal environmental technicians, other types of municipal personnel (gardener, police, councilor, etc.) and pest control technicians from the concessionary companies. This is the reason why, in order to get a standard outcome made by different people, in the week prior to the initial placement of ovitraps, a two-day seminar was arranged to address issues about *Ae. albopictus* biology and associated health matters, sampling and control methods, program arrangement, etc. The seminar content is available online, at <https://casiopea.um.es/cursospe//vigilanciaentomologicaii.f>.

All the standardized material, such as ovitraps, paddles, rabbit dry food and zip bags with printed identification labels (point, trap and sampling number), was provided to municipalities.

Alike *Aedes* spp. morphology eggs were identified under stereoscopic microscope and hatched in the laboratory, until adulthood, to verify their identity. Due to the big number of positive samples, not all mosquito eggs were reared to adulthood, although several samples from every positive point were always reared, both at the beginning and at the end of the season. There is a low possibility to find *Aedes echinus* (Edwards, 1920) or *Aedes geniculatus* (Olivier, 1791) [24], but we have never found them in the past years. The identification was carried out at the University of Murcia.

### 2.3. Study Period

The sampling period began on April 16th (week 16th of 2019), and the last sample was collected on November 26th (week 48th). A bi-weekly sampling was scheduled, so that 16 regional samplings were carried out. Samples were collected on the Tuesday of the first week of the corresponding bi-week, but sample collection in large municipalities could last two or three days. Therefore, the time unit was the bi-week instead of the specific collection date. The municipality of Murcia, in accordance with its own plan, carried out a more irregular sampling, not in line with the regional program, with an average interval of 12 days. Thus, despite starting one month later, it also carried out 16 samplings. These data were incorporated to regional scheme, according to the provided sample dates.

### 2.4. Intensity in Positive Places

On the one hand, the intensity of *Ae. albopictus* presence in positive municipalities was evaluated by a positivity index: percentage of bi-weekly positives (paddles with any egg) in the study period. On the other hand, the number of eggs by positive sample was taken into account. This is slightly controversial because, although the number of eggs is usually positively correlated with population density [7,25,26], this correspondence is not always exact [27]. This issue is more detailed in the next methodology subsection.

### 2.5. Phenology

Phenology represents population variation over time. As explained above, when ovitraps are used, there is no exact correspondence between the number of eggs and the real population. *Ae. albopictus* females exhibit skip oviposition behavior, which means that they spread eggs if containers with water are available. However, laying behavior varies throughout the season, and this pattern is more common in spring than in autumn [23]. In addition, the density of mosquitoes affects oviposition, because females prefer containers with a medium amount of con-specific (that is, a good breeding site where competitors are still scarce) [28,29]. For this reason, we prefer to use the term pseudo-phenology when the temporal variation of population density is measured in eggs from ovitraps [7]. In addition, the phenological evolution of positivity was also calculated, measured both as % positive points and as % positive municipalities over time. Both temporal patterns, for eggs and positivity, were compared as explained in the next subsection.

### 2.6. Statistical Analysis

Several statistical analyses were carried out in Rstudio v.1.2.5033 (R v.3.6.2) (Integrated Development for R. RStudio, PBC, Boston, MA, USA). The asymmetry of paired samples from double ovitraps by point was analyzed, in order to detect wrong placements resulting in lesser collections in one of two. The Friedman test for two-paired samples was used because the normality requirement was not met. Making a type I error and rejecting part of the right data was preferred, instead of misidentifying two traps of a point as correct (make a type II error) and accepting as right traps those which were not working properly. Thus, the significance level used was 90% instead of 95%. Moreover, correlation between average number of eggs vs. percentage of positive points and municipalities (positivity) was checked by using Spearman’s coefficient (the normality requirement was not met).

### 2.7. Dissemination of Results and Municipal Participation

After each sampling, the material obtained was studied, and the corresponding bi-weekly report was prepared. The general summary included the municipalities participating in that sampling, the number of samples collected and the points studied, as well as the percentage of losses and positives. It was followed by the list of all points in the program, with negative or positive results (blank if there were no samples from the specific point). The information from the municipality of Murcia was also reported, as a resumen of positive and negative points, but not included in the general counts, as it did not follow the same sampling pattern.

## 3. Results

### 3.1. Samples Study

A total of 6683 samples were obtained, which means 88.33% of the 7566 initially expected. In fact, the 11.67% missing did not correspond to usual losses due to stochastic causes; instead, an important reason for missed data was the incorrect or incomplete participation of several municipalities: lack of personnel during vacation periods or confusion of dates by technicians, not all municipalities began at the start of the plan, others canceled in advance and some did not participate totally or partially (supplementary data). Five municipalities were considered as non-participant: Aledo, Lorquí, Moratalla, Puerto Lumbreras and Villanueva del Río Segura. Moratalla never sampled, and the remaining four only made one or two samplings (see Supplementary Materials for data). Only one of them, Villanueva del Río Segura, was positive with scarce samples. Finally, in September, an extraordinary Mediterranean weather phenomenon, known as *“isolated depression at high levels”* (DANA, in Spanish), occurred: a very torrential storm which caused floods in many parts of the region [30]. It was an unusual event which prevented sample collection or swept samples along, either totally o partially, in several municipalities, resulting in a fall of samples in sampling 11th (see Supplementary Materials for data).

Thus, the real random loss (excluding the abovementioned issues) is reduced to 4.33%, and only 301 samples of the 6954 actually expected were lost. Duplication of ovitraps at sampling sites achieved a lesser loss of information per point and sampling, resulting in an overall loss of information of 38 data points (1.26%) of 3019 information/points expected during the study period, while the municipality of Murcia had a 6.14% loss derived from having single traps.

In total, 2639 of 6683 samples (34.88%) were positive, which corresponds to 229 positive points of 266 (86.09%), in 39 positive municipalities of 45, at any moment of the study period. When eliminating non-participating municipalities, 39 municipalities of 40 were positive, and 227 of 254 points were positive (89.37%). Most municipalities (72.5%) had positives in all points in some sampling, although density or positivity, as shown below, are more variable. Only the municipality of Bullas remained negative, although it was positive in 2018 and 2016 (unpublished data). The presence of *Ae. albopictus* was recorded for the first time in Abarán, Cehegín, Fortuna and Villanueva del Río Segura in 2019. In addition, *Ae. albopictus* was recorded for the first time in 2018 (unpublished data) and was confirmed in 2019 in Blanca, Calasparra, Caravaca, Cieza, Jumilla, Mula, Ojós, Pliego, Ricote, Santomera, Totana, Ulea and Yecla.

**Figure 1:**
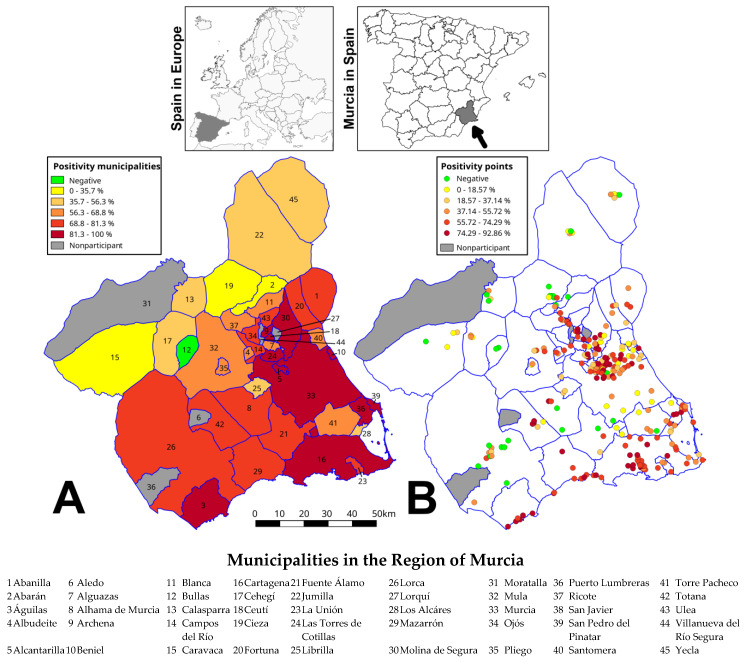
Positivity in 2019. (**A**) Municipalities (the numbers are related to the table of municipality names). (**B**) Sampling points. In each case, the Jenks natural breaks were calculated according each dataset. In red, the related municipalities to dengue cases in 2018, in the names list.

The highest positivity values occurred in the municipalities of the southern half of the region. Águilas, Cartagena and Murcia reached 100% of positive samplings (Figure 1A), although positivity, in general, is not uniform among these municipalities, but variable depending on the sampling points (Figure 1B).

Certain municipalities with very high-density sampling points were identified through the egg-count process. Águilas showed the highest average (353.1 eggs), and the average values at some of its points were among the ten highest: AGU05 (653.1 eggs), AGU02 (422.6 eggs) and AGU03 (354.6 eggs). In addition, this municipality had the paddle with the highest amount of eggs (point AGU05 with 2707 eggs in sampling sixth).

Furthermore, the egg count allowed us to study the asymmetry of the paired samples by point, in order to detect possible constant differences between them. Thus, using the Friedman test for two-paired samples, 30 points were identified where one of the traps constantly had significantly lower captures than the other (*p* < 0.10) due to undetectable causes.

### 3.2. Phenology

**Figure 2:**
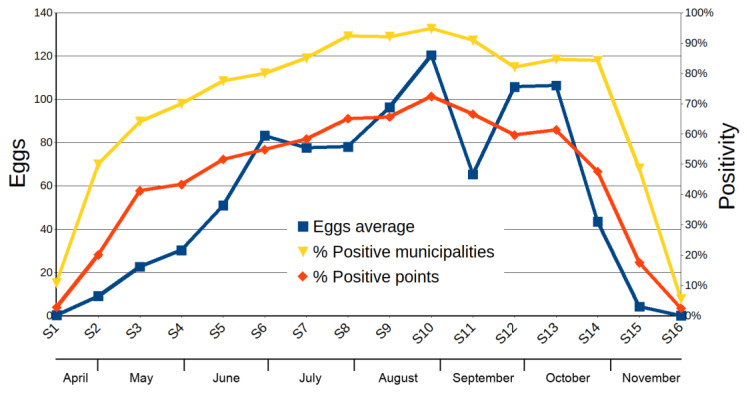
Fenology of *aedes albopictus* in the Region of Murcia, in 2019.

Figure 2 shows the pseudophenology by eggs and the positivity temporal variation per point and municipality.

The phenology of both parameters, eggs account and positivity, was similar in terms of the rise and fall pattern of values and the situation of the maximum in sample 10th, corresponding to the bi-week August 20th to September 3rd. The regressions eggs~% positive points and eggs~% positive municipalities (R² = 0.81, *p* < 2.2 × 10^−16^) showed that both parameters significantly fitted over time. In sampling 11th, a drop was observed, but it could be an artifact, as the abovementioned weather phenomenon (DANA) may have caused an immediate population drop, but, simultaneously, there was a significant loss of samples (even whole municipalities) (see Supplementary Materials data).

### 3.3. Dissemination of Results and Municipal Participation

The samples were quickly studied, and the bi-weekly reports were always available before the next sampling. They were made public in the URL https://www.um.es/grzba/Vigilancia_Mosquito_Tigre/. In this way, each municipality had all the information with regard to the degree of participation and the positive municipalities and points. The intention of this dissemination was to motivate involvement and, in relation to the specific information, to serve as a tool in active municipal management. Thus, technical consultations also took place when new positive points appeared in areas or municipalities that had been free of *Ae. albopictus* so far.

## 4. Discussion

The participation of municipalities was high in 2019, both in terms of the number of municipalities of the region and the study period. However, the non-participant municipalities represent a lack of information that should be solved in the future, in order to avoid public health uncertainty. In previous years, some of them, such as Aledo (2018) and Lorquí (2016, 2018), were positive, while others, including Moratalla (2018), Puerto Lumbreras (2014, 2016, 2018) and Villanueva del Río Segura (2015, 2016 and 2018), were negative [7,31] (and unpublished data of 2018). Of these three previously negative municipalities, only Villanueva resulted in being positive in 2019 (the first record). However, the previous samples of the remaining two were scarce in the past and also in 2019. As a result, their negativity cannot be ensured. Thus, *Ae. albopictus* was recorded in all sufficiently studied municipalities in the Region of Murcia, which updates the last published known distribution [8].

The minimum necessary number of ovitraps can be calculated by the Taylor equation [32], as recommended or previously done in other studies [25,33,34,35]. In our case, as the purpose of the regional surveillance is to act both as a way of monitoring the environment health service and as a management tool for municipalities, the arrangement of the sampling points was driven by two factors, the limited workforce and the idea of getting coverage over municipalities’ urban/suburban areas. In relation to the first one, we were able to study all the samples on a bi-weekly basis, to get results prior to the next sampling, so that we could increase the number of points/samples every bi-week. With respect to urban/suburban coverage, rather than calculating the minimum necessary number of traps, we tried to cover urban/suburban areas, estimating the action range in about 300 m diameter, according to studies on the dispersal of *Ae. albopictus* females [36,37]. Thus, if full participation of municipalities was achieved, the program could get to cover a high percentage of urban areas in the region.

Regarding egg counting, in addition to estimating mosquito population density, the study of paired ovitraps asymmetry has an operational usefulness in detecting ovitraps misplacements. The causes, which were undetected during placement, can be varied, including placement under an unidentified repellent plant, insufficient shade, excessive wind, etc. In case of constant deficiencies in one of two, a slight displacement from the defective location would be recommendable.

From the health perspective of entomological surveillance, both positivity and account of eggs provide clues about the risk areas, both in regard to reaction to bites and arbovirosis transmission. Due to the fact that vectorial capacity is influenced by vectors abundance [26,38], it is obvious that points with a very high count of eggs result in mosquito bites, since it means high vector population density [23]. However, throughout the years that we have been working with several municipalities, we have observed that bite complaints are more usually related to the constant presence of *Ae. albopictus* (high positivity) than to density (unpublished data). As pointed out in the results, positivity varies across the region, and, since it affects transmission risk, it should be taken into account as a health indicator. In addition, it could be useful to define risk areas when ovitraps are used, although more studies must be conducted. Codeço et al. [39] used a positivity index based on a spatial perspective, that is, the proportion of positive traps of the total units installed, whereas our study is grounded on a temporal basis. As it was shown in the results, the majority of the points included in the present work were positive at any time in 2019. Then, the spatial index would not be useful with our data because our study units, that is, municipalities, have few points. Excepting the four bigger ones, the rest of municipalities have got an average of 3.6 sampling points. In the same train of thought, phenological variation of positivity could be used to indicate the periods of greatest bite risk and, therefore, of greatest health consequences.

The dengue cases in 2018 were related to the municipalities of San Javier, Murcia (northern part) and Molina de Segura, but probably not to Alhama de Murcia (some patients were registered at this municipality, but they really resided in Murcia). All of these municipalities have been colonized by *Ae. albopictus* for years (between 2011 and 2014) and achieved high positivity in 2019 (Murcia: 100% positivity, 87.5% positive points; Molina: 96.6%, 100%; San Javier 93.8%, 100%, Alhama: 75%, 50%). Two of them, Murcia and Molina de Segura, would represent high-risk areas because both have high positivity values and high human population and density (Murcia: 453,258, 511.6 pop./km²; Molina: 71,890, 424.1 pop./km²) [17]. Other municipalities with similar characteristics, although unrelated to the 2018 cases, are Cartagena (100% positivity; 100% positive points; population 214,802, density 384.9 pop./km²) and Alcantarilla (87.5%; 100%; 42,048, 2588.5 pop./km²).

Finally, in any epidemiological surveillance program, *feedback is vital in order to promote involvement and improved quality of data* [40]. As the WHO manual for planning social mobilization for dengue control pointed out [41], *the loop connecting research, decisions, and actions* must be completed, disseminating the results of the research so that they can be used for management, logistics, surveillance, vector control, etc. In this way, these were our intentions with the real-time information disseminated by the bi-weekly reports: providing feedback to promote involvement, as well as a tool, for municipal mosquito control. In fact, many of the municipal representatives informed us about their feeling of being actively implied in the surveillance.

## 5. Conclusions

*Ae. albopictus* was collected in almost all the studied municipalities of the region in 2019, but the samples showed different spatial and temporal intensities. Thanks to the standardization of the sampling method and the synchrony of samplings, a better global analysis was possible, and the regional results were properly compared.

Although several municipalities failed to participate, the majority of the municipalities of the Region of Murcia took an active part of the regional program of entomological surveillance. Consequently, it can be concluded that the program is appropriately established. Municipal participation must be promoted in order to recover non-participant municipalities and avoid preventable sample losses which are not due to chance.

The use of paired traps is a great advantage, as it significantly reduces information loss while helping to correct erroneous positions that generate false negatives. Positivity could be used to define risk areas and periods.

After verifying that the identification of bi-weekly samples and data analysis workload are assumable, the number of sampling points could be increased to cover the urban/suburban areas of the region more exhaustively.

## Figures and Tables

**Table 1:** Number of sampling points per municipality.

Municipalities	Points	Municipalities	Points	Municipalities	Points
Abanilla	3	Cartagena	36	Moratalla	2
Abarán	3	Cehegín	3	Mula	4
Águilas	6	Ceutí	2	Murcia	58
Albudeite	2	Cieza	7	Ojós	2
Alcantarilla	7	Fortuna	4	Pliego	2
Aledo	2	Fuente Álamo	4	Puerto Lumbreras	4
Alguazas	2	Jumilla	4	Ricote	3
Alhama de Murcia	4	La Unión	3	San Javier	6
Archena	3	Las Torres de Cotillas	4	San Pedro del Pinatar	4
Beniel	2	Librilla	2	Santomera	3
Blanca	2	Lorca	16	Torre Pacheco	6
Bullas	2	Lorquí	2	Totana	5
Calasparra	4	Los Alcázares	3	Ulea	2
Campos del Río	2	Mazarrón	6	Villanueva del Río Segura	2
Caravaca	4	Molina	13	Yecla	6

## Supplementary Materials

The following are available online at https://www.mdpi.com/1660-4601/17/11/4173/s1, Table S1: Samplings carried out by each municipality for *Aedes albopictus*, in 2019, in the Region of Murcia.
