# Supplementary material for: Consolidation of Aedes albopictus Surveillance Program in the Autonomous Community of the Region of Murcia, Spain
Source: Int J Environ Res Public Health. 2020 Jun 11;17(11):4173. doi: 10.3390/ijerph17114173 (PMC7312822; doi:10.3390/ijerph17114173)
Supplement: Supplementary file 1 [file ijerph-17-04173-s001.pdf]

Supplementary data

Table S1: Samplings carried out by each municipality for *Aedes albopictus,* in 2019, in the Region of Murcia. *1: Positive sampling; 0: Negative sampling; Blank: no sample*s.

Five municipalities were considered as nonparticipant: Aledo, Lorquí, Moratalla, Puerto Lumbreras and Villanueva del Río Segura. Moratalla never sampled and other four only made one or two samplings. Nonparticipant municipalities are stressed in italics.

**Municipalities**	**S1**	**S2**	**S3**	**S4**	**S5**	**S6**	**S7**	**S8**	**S9**	**S10**	**S11**	**S12**	**S13**	**S14**	**S15**	**S16**	**N**	**Positives**
Abanilla	0	0	1	1	1	1	1	1	1	1	1	1	1	1	1	0	16	13
Abarán	0	0		0	0	0	0		1	1	0	1	1	1	0	0	14	5
Águilas	1	1	1	1	1	1	1	1	1	1	1	1	1	1	1	1	16	16
Albudeite	0	0	1	1	1	1	1	1	1	1	1	1	1	0	0	0	16	11
Alcantarilla	0	1	1	1	1	1	1	1	1	1	1	1	1	1	1	0	16	14
Aledo		0															1	0
Alguazas	0	1	1	1	1	1	1	1	1	0	1	1	0	0	0	0	16	10
Alhama de Murcia	0	0	1	1	1	1	1	1	1	1	1	0	1	1	1	0	16	12
Archena	0	1	1	1	1	1	1	1	1	1	1	1	1	1	1	0	16	14
Beniel		1	1	1	1	1	1	1	1	1		1	1	1		0	13	12
Blanca	0	1	0	0	0	1	1	1	1	1							10	6
Bullas	0	0	0	0	0	0	0	0			0	0	0	0	0	0	14	0
Calasparra	0	0	0	1	0	0	0	1	1	1	1	1	1	1	0	0	16	8
Campos del Río	0	1	1	1	1	1	0	1	1	1	1	1	0	1			14	11
Caravaca	0	0	0	0	1	0	1	0	0	0	0	0	1	1	0	0	16	4
Cartagena	1	1	1	1	1	1	1	1	1	1	1	1	1	1	1		15	15
Cehegín	1	0	0	0	0	0	1	1	1	1	1	0	1	0	0	0	16	7
Ceutí	0	1	1	1	1	1	1	1	1	1	1	1	1				13	12
Cieza	0	0	0	0	0	0	1	1	0	1		0	1	1	0	0	15	5
Fortuna			1	0	1	1	1	1				1	1	1		0	10	8
Fuente Álamo	0	1	0	1	1	1	1	1	1	1		1	1	1	1	0	15	12
Jumilla	0	0	0	0	0	1	1	1	1	1		1	1	1	0	0	15	8
La Unión	0	0	1	0	1	1	1	1	1	1	1	1	1	1	1	0	16	12
Las Torres de Cotillas	0	1	1	1	1	1	1	1	1	1	1	1	1	1	1	0	16	14
Librilla	0	0	1	1	1	1	1	0	0	1	1	0	0	0	0	0	16	7
Lorca	0	1	1	1	1	1	1	1	1	1	1	1	1	1	0	0	16	13
Lorquí		0		0													2	0
Los Alcázares	0	0	0	1	0	0	0	1	1	1	1	1	1	1	1	0	16	9
Mazarrón	0	0	1	1	1	1	1	1	1	1	1	1	1	1	1	0	16	13
Molina de Segura	0	1	1	1	1	1	1	1	1	1	1	1	1	1	1	0	16	14

Moratalla																	0	0
Mula	0	0	0	0	1	1	1	1	1	1	1	1	1	1	1	0	16	11
Murcia			1	1	1	1	1	1	1	1	1	1	1	1	1	1	14*	14
Ojós	0	1	1	1	1	1	1	1	1	1	1	1	1	1	0	0	16	13
Pliego	0	0	0	0	1	1	1	1	1	1	1	1	0	1	1	0	16	10
Puerto Lumbreras		0															1	0
Ricote	0	0	0	1	1	1	1	1	1	1	1	1	1	1	0	0	16	11
San Javier	1	1	1	1	1	1	1	1	1	1	1	1	1	1	1	0	16	15
San Pedro del Pinatar	0	1	1	1	1	1	1	1	1	1	1	1	1	1	1	0	16	14
Santomera	0	1	1	1	1	1	1	1	1	1	1	0	0	0	0	0	16	10
Torre Pacheco	0	0	0	1	1	1	1	1	1	1		1	1	1	0	0	15	10
Totana	0	1	1	1	1	1	1	1	1	1	1	1	1	1	0	0	16	13
Ulea	0	0	1	1	1	1	1	1	1	1	1	1	1	1	0	0	16	12
Villanueva del Río Segura		0		1													2	1
Yecla	0	1	0	0	0	0	0	1	1	1	1	1	1	1	0	0	16	8
**Participant municipalities**	37	42	39	42	40	40	40	39	38	38	33	39	39	38	35	36		
**Positive municipalities**	4	19	25	29	31	32	34	36	35	36	30	32	33	32	17	2		

* Samplings of the municipality of Murcia have been adjusted to the bi-weekly sequence of the program, according to their sampling dates. Although this municipality carried out 16 samplings, they were collected in a 12-day median interval, therefore only 14 bi-weekly periods were covered.
